# Metabolic pattern formation in the tumor microenvironment

**DOI:** 10.15252/msb.20167518

**Published:** 2017-02-09

**Authors:** Ziwei Dai, Jason W Locasale

**Affiliations:** ^1^ Department of Pharmacology and Cancer BiologyDuke UniversityDurhamNCUSA

**Keywords:** Cancer, Quantitative Biology & Dynamical Systems, Signal Transduction

## Abstract

Metabolic alterations including increased glycolysis are a common feature of many cancers. In their recent study, Lowengrub, Waterman, and colleagues (Lee *et al*, 2017) report a spatial pattern of glycolysis in solid tumors that occurs within the tumor microenvironment. This spatial organization is linked to gradients derived from Wnt signaling and nutrient availability that mediate a reaction‐diffusion mechanism and is consistent with a Turing‐type model of spatial localization.

It has long been known that tumor cells exhibit the Warburg effect; that is, there is enhanced glucose uptake and fermentation to lactate even with a sufficient supply of oxygen (Dai *et al*, 2016; Liberti & Locasale, 2016). It is also well appreciated that there is considerable intratumor heterogeneity in cellular metabolism with different cells within the same tumor having different glycolytic rates (DeBerardinis & Chandel, 2016). Much of this heterogeneity is thought to result from differences in nutrient accessibility, which is supported by a correlation between nutrient utilization and the degree of perfusion in certain tumors (Hensley *et al*, 2016). Thus, it is largely believed that metabolic heterogeneity is in large part determined by the location of the tumor cell relative to the vasculature (Sonveaux *et al*, 2008). In a recent study (Lee *et al*, 2017), a distinct spatial pattern of cells exhibiting the Warburg effect was observed in colorectal tumors and this spatial localization was attributed to the coupling of gradients in diffusible activators and inhibitors of Wnt signaling with nutrient availability.

By performing immunostaining to monitor the activity of phosphorylated PDH (pPDH), used as a marker for the Warburg effect, and LEF‐1, used as a surrogate of Wnt signaling activity, Lee *et al* (2017) observed a spotted clustering of cells with high pPDH and high LEF‐1 levels in both xenograft models and primary colorectal tumors (Fig 1A). Further comparison of the pPDH and LEF‐1 patterns showed similar spatial distributions, suggesting a connection between Wnt signaling and metabolic pattern formation. Additional support for this finding is contained in previous literature that notes a connection between Wnt signaling and the Warburg effect (Esen *et al*, 2013).

**Figure 1:**
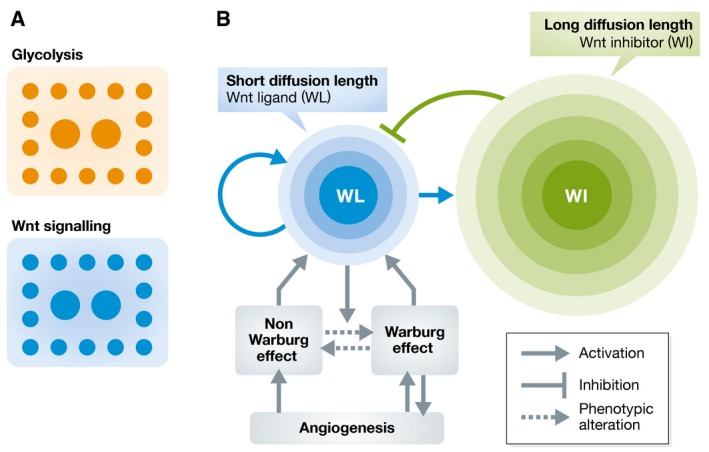
Patterning of glycolysis in tumors (A) Observation of correlated patterning of glycolysis and Wnt signaling in tumors. Smaller clusters are located near the boundary determined by the margin of the tumor and normal tissue regions. (B) Reaction‐diffusion mechanism resulting in a Turing‐type metabolic pattern. The radius of the circles shows the length scales of diffusion for the Wnt ligand (WL) and Wnt inhibitor (WI), and the color represents the concentration level. The colored part of the diagram contains the essential features of the reaction‐diffusion model. The gray part of the diagram denotes the relationship to the Warburg effect and angiogenesis in the model.

The Wnt signaling pathway has an important role in tumorigenesis (Polakis, 2000). Previous research has also linked Wnt signaling to pattern formation in development; for example, formation of regular patterns of hair follicles is regulated by Wnt signaling via a reaction‐diffusion (RD) system that exhibits a Turing‐type mechanism (Sick *et al*, 2006). The essential feature of these RD mechanisms is the interaction between an activator with a shorter diffusion length scale and an inhibitor with a longer diffusion length scale (Kondo & Miura, 2010), which enables the formation of a stationary periodic pattern (Fig 1B), or Turing pattern, named after Alan Turing. Inspired by the well‐established theory of RD mechanisms, the authors constructed a Turing‐type model combining metabolic phenotypes regulated by Wnt signaling, cell turnover, nutrient delivery, and angiogenesis mediated by glycolytic cells to simulate the formation of a Warburg effect or glycolytic pattern. The feasibility of such a model was first confirmed by simulating the spotted clusters that resemble patterns observed from the immunostaining of the tumors.

The inclusion of both Wnt signaling and glycolytic patterns in the mathematical model allows for a deeper characterization of how Wnt signaling could affect spatial patterning of metabolism in tumors. For instance, the model indicated that simply decreasing the level of Wnt signaling leads to a reduced number of glycolytic cells without affecting the location of the overall pattern. However, interfering with Wnt signaling by expressing a dominant‐negative LEF‐1 (dnLEF‐1) or a dominant‐negative TCF‐1 (dnTCF‐1) in xenografts resulted in both reduction in glycolysis activity and alterations in metabolic patterning that was marked by formation of larger and sparser clusters of glycolytic cells. In order to resolve this discrepancy, the authors hypothesized that the dnLEF‐1 and dnTCF‐1 models were eliciting effects more complicated than inhibiting Wnt signaling. Parameter sensitivity analysis of the model indicated that the change in spot distribution was likely to be the consequence of other factors that increase the diffusion length scale of both the activator and the inhibitor of Wnt signaling. One possible mechanism of increased diffusion length could be the enhanced expression of factors known as Wnt diffusers. By analyzing expression of candidate genes in dnLEF‐1 and dnTCF‐1 xenograft tumors, the authors identified the Wnt diffusers SPOCK2, GPC4, and SFRP5 as increased in response to interference of Wnt signaling, thus supporting the prediction of the model. Interestingly, increased expression of Wnt diffusers was also observed in tumor samples from colorectal cancer patients treated with radio‐ and chemotherapy, suggesting that increasing the diffusion length of Wnt signaling molecules might be a general strategy for cancer cells to respond to stress. This would provide an example of the role of the physical properties of a tumor in determining its phenotype. Finally, the model was further applied to simulate therapeutic interventions in tumors with metabolic heterogeneity and it demonstrated potential synergy between inhibiting Wnt signaling and selectively targeting glycolytic cells.

As the authors mention, it is not entirely possible to exclude alternative models that could produce similar outcomes and it is also unclear whether pPDH is an actual measure of glycolytic metabolism and thus metabolic heterogeneity. Nevertheless, the study by Lee *et al* (2017) identified relationships between reaction‐diffusion mechanisms and metabolic pattern formation, thus providing a framework to understand metabolic heterogeneity within tumors. It indicates that despite the enormous apparent complexity of the tumor microenvironment, intratumor metabolic heterogeneity in some cases could be the consequence of a simple, self‐organized process explained by only a few essential features contained within a general mechanism. Future research is needed to determine whether metabolic pattern formation is beneficial for tumorigenesis or is a passenger (i.e. bystander) phenomenon resulting from the coupling of Wnt signaling and nutrient availability. Whether similar metabolic patterns exist in other tumor types that do not rely on aberrant Wnt signaling is also a further area of investigation. Another unexpected finding is that tumors appear to respond to treatments by increasing the diffusion length scale of Wnt signaling molecules even if the treatments do not target Wnt signaling directly, suggesting that the diffusion of signaling molecules could have some roles in tumor maintenance. How such a mechanism is established is unknown and worthy of future study. Nevertheless, the study of Lee *et al* (2017) provides an elegant example of the utility of mathematical models to provide a framework for understanding metabolic heterogeneity in the tumor microenvironment.
